# Evaluating Ergonomics Training in Ophthalmology Residency: A Pilot Study

**DOI:** 10.7759/cureus.103081

**Published:** 2026-02-06

**Authors:** Matthew B Urban, Brandon M Bessen, Bentzion Kleiman, Avery Morrison, Savannah Kumar, Alicia Jiang, Elizabeth Drugge, Kelly Hutcheson, Abha Amin

**Affiliations:** 1 Ophthalmology, New York Medical College, Valhalla, USA; 2 Ophthalmology, Bascom Palmer Eye Institute, University of Miami, Miami, USA; 3 Ophthalmology, Northwell Health, Great Neck, USA; 4 Ophthalmology, Westchester Medical Center, Valhalla, USA; 5 Public Health, New York Medical College School of Health Sciences and Practice, Valhalla, USA

**Keywords:** career longevity, ergonomics, graduate medical education, ophthalmology residency, ophthalmology training, resident education

## Abstract

Background

Slit lamp examinations (SLEs) require sustained visual focus and precise positioning, often performed repeatedly during clinical encounters. Without formal ergonomics training, residents may develop poor posture habits early in training. Targeted educational interventions during residency may offer an opportunity to promote safer examination techniques, support occupational health, and strengthen healthcare system safety by reducing provider injury risk and practice disruption. This study aimed to evaluate the short-term effect of a structured ergonomics education protocol on the posture of ophthalmology residents performing SLEs.

Methodology

In this single-center pilot quality improvement study conducted from July 27, 2023, to February 28, 2024, resident posture was assessed during SLEs using the Rapid Entire Body Assessment (REBA) scale, where higher scores indicate greater musculoskeletal risk. Seven ophthalmology residents from Westchester Medical Center were recruited for the study. Residents were evaluated at baseline and randomly assigned to either a control group (n = 3) or an intervention group (n = 4). The intervention group completed an American Academy of Ophthalmology-derived ergonomics training program, while the control group received no additional training. Post-intervention REBA scores were collected and analyzed using a linear mixed regression model accounting for repeated measures, with statistical significance defined as α of 0.05.

Results

A total of 143 SLEs (n = 143 patients) were included in our study. Mean REBA scores significantly decreased in the intervention group (n = 85 SLEs, p < 0.0001), while no significant change was observed in the control group (n = 58 SLEs, p = 0.4).

Conclusions

An ergonomics education intervention was associated with improved resident posture during SLEs. These findings support the feasibility and potential value of incorporating structured, low-cost ergonomics training into ophthalmology residency curricula. This intervention may also be transferable to other procedural specialties seeking to promote occupational health and sustainable practice habits during postgraduate medical training.

## Introduction

Ergonomic risk

The healthcare sector has among the highest rates of occupational injuries compared to other private industries, with over 900,000 cases documented in 2023 [1]. Occupational injuries in healthcare range from rare incidents, such as needlestick injuries, to more frequent musculoskeletal (MSK) injuries that often result in chronic disorders. Surgeons face an increased risk of developing chronic musculoskeletal disorders (MSDs) due to the unique postural demands of surgery, which often involve physically awkward and sustained positions. Conditions such as carpal tunnel syndrome, cervical and lumbar spine pathologies, rotator cuff injuries, tendonitis, and trigger finger are prevalent [1,2]. These conditions result from the repetitive use of non-neutral body mechanics (prolonged flexion, extension, or overhead reaching), which gradually place strain on the MSK system.

MSK pain, discomfort, and injury are likewise significant risks when practicing ophthalmology. Over 50% of ophthalmologists worldwide suffer from work-related MSK pain, and up to 8% require spinal procedures to manage pain and symptoms [3,4]. A recent meta-analysis showed that this rate is comparable to other surgical subspecialties, where 7.6-10.7% of physicians undergo surgical intervention for spine-related disorders. The study also reported that 12% of surgeons with work-related MSDs experienced significant career impact, including leave of absence, practice restriction, or early retirement, underscoring the substantial disability burden across surgical fields [5].

Honavar et al. outlined the major work-related MSDs experienced by ophthalmologists, which include back and neck pain, numbness, and carpal tunnel syndrome [6]. These MSDs are often attributable to repetitive movements during slit lamp examinations (SLEs), indirect ophthalmoscopy, and surgical procedures. MSDs may also result from incorrect posture during SLE. Ideally, the head, neck, and torso should remain vertically aligned in a neutral posture; however, ophthalmologists frequently lean toward the slit lamp, displacing the neck out of alignment and placing cumulative stress on the cervical spine [6].

Individual factors such as gender, height, and body habitus influence positioning at the slit lamp and other ophthalmic equipment [6]. For example, shorter practitioners may need to lean forward excessively, while taller practitioners may be forced into sustained neck flexion, both of which increase ergonomic strain. These anthropometric variables contribute to the heterogeneity of MSK risk among ophthalmologists and should be considered in ergonomic assessment and intervention [7].

Due to the inherent MSK risks in ophthalmology, many practitioners resort to pain medication, reduce clinical hours, and retire early [3,4]. One study found that 14% of ophthalmologists intend to leave practice prematurely due to pain and spinal injuries [7]. While based on a different study design, this proportion is comparable to the ~12% disability-related career impact reported in other surgical subspecialties [5]. Despite the prevalence of these issues, a substantial number of ophthalmologists remain unaware of the daily MSK risks they face. Provider ergonomics is not only an issue of individual occupational health but also of healthcare system safety, as work-related MSK injury contributes to reduced clinical capacity, procedural limitations, and early workforce attrition [5,7].

How to evaluate ergonomic risk

The Rapid Entire Body Assessment (REBA) tool, used in studies by Raman et al. and Morrison et al., evaluates posture-related risks in clinical settings [8,9]. The REBA tool allows for a holistic score of MSK risk by systematically evaluating the MSK strain on each body segment. Designed by experts across multiple health disciplines, it considers both movement and still posture, along with how workers interact with tools, such as grip strength and equipment safety, through its Coupling Score. REBA is applied to photos of healthcare workers while performing procedures, enabling the user to stratify risk through this scoring technique [10].

REBA has been effectively employed in assessing the impact of an education protocol. In a study performed on 74 operating room nurses in Iran, Abdollahi et al. used the REBA scale to classify the nursing staff by risk of MSD. As determined by their score, nurses were stratified into low risk (13.5%), medium risk (51.4%), or high risk (35.1%) groups. The data these researchers collected was later used to design an ergonomics educational program that showed a largely significant reduction in the risk of MSDs as a result of the program [11].

Ergonomic solutions

The increasing prevalence of MSK pain in ophthalmology has led to efforts to improve occupational health, including a new focus on improving ergonomics to mitigate injury [6,7]. Some solutions include yoga and stretching exercises, education, and awareness of MSK risk [12-15]. The American Academy of Ophthalmology (AAO) task force offers online courses for best practices in the workplace for ophthalmologists, which could improve resident posture as well if incorporated into ergonomics training protocols [14,16-18]. The results of a Canadian study in 2019 found improvement in injury risk following an educational module on ergonomics for SLE [4]. These findings motivated our team to develop an ergonomics education protocol derived from that of the AAO. Our study aims to investigate the impact of early ergonomic education in a US ophthalmology residency program. We intend for our findings to increase awareness of ergonomic risk and inform solutions to prevent long-term MSK injury.

## Materials and methods

Study design

This single-center pilot quality improvement study from July 27, 2023, to February 28, 2024, quantifies the impact of ergonomic education on the posture of residents performing SLEs using the REBA scale, where higher REBA scores indicate greater risk of MSK injury. This study was granted exemption by the Institutional Review Board of New York Medical College (approval number: 20183).

Study population and inclusion/exclusion criteria

We conducted this study at Westchester Medical Center (WMC) by recruiting seven ophthalmology residents. Four residents were assigned to the intervention group, and three residents were assigned to the control group. Residents were followed longitudinally over seven months.

Patients were recruited through voluntary consent in the resident ophthalmology clinic at WMC. All SLEs performed on patients aged 12-80 were eligible for inclusion. Only examinations conducted using the standard ophthalmic chair were included in the analysis. All examinations were performed using the same slit lamp model and standard ophthalmic chair within the resident clinic to reduce equipment-related variability.

Data collection

Seven ophthalmology residents from WMC were observed during an entire outpatient clinic session, defined as the full list of patients scheduled for that day. Residents were aware that posture assessment would be assessed as part of the study, but were not informed which patient encounters would be included in the analysis.

Photographs were taken during SLEs of patients who voluntarily consented to participate. Examinations of non-consenting patients were still observed clinically, but not photographed or included in image-based analysis. Baseline photographs were subsequently evaluated using the REBA scale to quantify ergonomic risk. Image acquisition followed a standardized protocol with predefined anterior, posterior, and lateral viewpoints at a consistent distance and height relative to the slit lamp to improve scoring reliability.

Ergonomic analysis

The REBA is a validated ergonomics scoring tool that holistically evaluates MSK posture to estimate the risk of developing MSDs. Given the inherent cervical spine and lumbosacral strain associated with SLEs, REBA provides an appropriate framework to evaluate resident posture and assess the efficacy of our ergonomics intervention.

REBA scores range from 1 to 15, with higher values representing greater MSK risk [7]. A baseline REBA score for an SLE is typically 2-3, and any value above this indicates increased ergonomic risk [9]. Researchers assessed resident posture, body torsion, and vertebral alignment to generate REBA scores. For each SLE encounter, three standardized photographs (anterior, posterior, and lateral) were obtained, and scores were calculated based on posture, force requirements, and movement type.

To minimize inter-rater variability in REBA scoring, each encounter was independently scored by three observers. The scores were then averaged, and the mean value was used as the REBA score for each encounter. Training for each observer occurred over three weeks, with a minimum of four training hours in the clinic per week. This consisted of simulated encounters where observers could practice taking photographs during SLEs, perform scoring according to the REBA scale, and ensure accurate scoring by cross-referencing the standards of the REBA scoring system [10].

To limit the Hawthorne effect, where residents may consciously alter posture when aware of observation, a random subset of encounters was included in the analysis. Although observers were present during all SLEs to obtain photographs, residents were unaware which patients had consented to study participation. This approach partially mitigated observer-related behavior changes. Additionally, for patients with multiple SLEs during the study period, only the initial encounter was analyzed to preserve randomization integrity and prevent duplicate representation.

Training protocol

Following baseline measurement, four residents underwent an ergonomic training protocol adapted from the AAO ergonomic task force. The hour-long program consisted of the following three parts: videos on ergonomic techniques for ophthalmologists, yoga and stretching exercises, and a personalized analysis of posture tendencies and how to address them [12,13,18,19]. All training sessions were completed in the sixth week of a three-month rotation. Repeat REBA measurements were assessed four weeks after intervention to evaluate the impact of ergonomics training on posture.

The educational intervention included standardized AAO-derived modules covering slit lamp positioning, neutral spine alignment, equipment height adjustment, and examiner-patient positioning [14,16,18]. Stretching instruction emphasized cervical, thoracic, lumbar, and shoulder mobility [12]. Personalized feedback followed a structured checklist evaluating head tilt, neck flexion, trunk lean, shoulder elevation, and wrist positioning to ensure consistency across participants [4,6,10].

Statistical analysis

A linear mixed-effects regression model was fit to examine the relationship between the REBA score and treatment group across timepoints. “Resident” was included as a random effect to account for clustering of repeated SLEs within individual residents, and the model assessed baseline differences in REBA scores between groups. In the presence of a significant treatment-by-time interaction, pairwise comparisons between predictor levels were conducted with Bonferroni correction to maintain a familywise type I error rate of 0.05. All hypothesis tests were two-sided, and statistical significance was defined as p-values <0.05. Analysis was conducted using R version 4.4.2. The lme4 and lmerTest packages were used to fit and obtain estimates from the linear mixed-effects regression model.

## Results

The final sample size for this study was 143 SLEs (n = 143) performed on 143 individual patients (n = 143) by seven ophthalmology residents. The SLEs included in our study were performed by seven ophthalmology residents at WMC. This sample size was a result of the class size of ophthalmology residents at WMC (three residents per year, nine total ophthalmology residents). Our team successfully recruited seven of the nine residents at WMC, representing the total number of residents who rotated through the WMC clinic during the study period. Once recruitment was complete, an automated randomizer assigned residents to the intervention or control group.

Table 1 includes the descriptive details of the seven ophthalmology residents from WMC who were included in our study. Four were female, and three were male. In addition, three residents were in their final postgraduate year (PGY-4), two residents were in postgraduate year three (PGY-3), and two residents were in postgraduate year two (PGY-2). Baseline REBA score, experimental REBA score, and number of SLEs are included for each resident. Treatment and control group assignment is also labeled. Lastly, the height of each resident is reported in Table 1 in inches (range = 60-73, mean = 65.93).

**Table 1:** Descriptive details of WMC ophthalmology residents. WMC = Westchester Medical Center; REBA = Rapid Entire Body Assessment; SD = standard deviation

Control vs treatment group	Resident ID/Gender	Postgraduate year (PGY)	Baseline REBA (SD)	Post REBA (SD)	Height of residents (inches)
Treatment	Female #1	PGY-4	5.23 (0.92)	4.82 (0.67)	64
Treatment	Female #2	PGY-4	4.58 (1.12)	3.78 (0.69)	60
Treatment	Female #3	PGY-3	4.81 (0.78)	4.24 (0.42)	60
Treatment	Male #1	PGY-2	4.00 (0.54)	4.08 (0.32)	69
Control	Female #4	PGY-2	4.25 (0.53)	4.11 (0.93)	64.5
Control	Male #2	PGY-4	3.75 (0.49)	4.02 (0.61)	73
Control	Male #3	PGY-3	4.69 (0.89)	4.87 (0.90)	71

The number of SLEs and the average REBA score are included in Table 2; data are stratified by treatment group and time point (pre-intervention vs. post-intervention). The control group of three residents performed a total of 58 SLEs (n = 34 pre-intervention, n = 24 post-intervention). The intervention group of four residents performed a total of 85 SLEs (n = 44 pre-intervention, n = 41 post-intervention). REBA scores are reported as mean values, and post-intervention values were recorded four weeks after training. The control group's pre-REBA score was 3.91, while post was 4.08. In the intervention group, the average pre-training score was 5.06, with an average post-training score of 4.14. Average REBA scores decreased across the trained cohort of four residents (n = 85 SLEs, β = 0.97, 95% confidence interval (CI) = 0.60 to 1.4; t = 6.95, p < 0.0001), but did not change in the untrained control group of three residents (n = 58 SLEs, β = -0.20, 95% CI = -0.67 to 0.26; t = -1.18, p = 0.4).

**Table 2:** Average REBA score stratified by treatment group and grouped by time point. REBA = Rapid Entire Body Assessment; SD = standard deviation; SLEs = slit lamp examinations

	Control (n = 3)		Intervention (n = 4)	
Time point (# of SLEs)	Pre (34)	Post (24)	Pre (44)	Post (41)
Average REBA, mean (SD)	3.91 (0.76)	4.08 (0.79)	5.06 (0.96)	4.14 (0.67)

The linear mixed-effects regression model identified a significant interaction between treatment group and time point. This indicates that within-resident changes in REBA scores over time differed significantly by treatment group after accounting for resident-level clustering (β = -1.2, 95% CI = -1.6 to -0.74; t = -5.29, p < 0.001). Within the control group, no significant change in the average REBA score was observed from pre- to post-intervention (p = 0.4). Before the intervention, REBA scores were significantly higher in the intervention group compared to the control group (β = -1.1, 95% CI = -2.2 to -0.05; t = -3.95, p = 0.02), reflecting a baseline imbalance in posture severity between groups. Post-intervention, no significant difference in REBA score was observed between the two groups (β = 0.03, 95% CI = -1.0 to 1.1; t = 0.109, p = 0.9).

## Discussion

We observed a statistically significant improvement in the average REBA score for those who received the ergonomic intervention training (p < 0.0001), whereas no improvement was demonstrated within the control group (p = 0.4). These findings indicate that brief, targeted ergonomic training was associated with greater within-resident improvement in posture compared with controls, after accounting for resident-level clustering. Although changes from pre- to post-intervention showed significant differences, post-intervention scores between groups were similar (control = 4.08 ± 0.79; treatment = 4.14 ± 0.67). This finding was likely influenced by higher baseline REBA scores in the intervention group, a consequence of the small cohort size. While a significant improvement in the average REBA score was only detected in the intervention group, the baseline variability suggests that the training protocol may have contributed to the observed differential effect. Our results do suggest, however, that the intervention did have a significant overall effect on REBA score. This observation aligns with prior studies in healthcare settings, which have demonstrated posture improvement following targeted ergonomics training, underscoring the importance of awareness and research in medical ergonomics [4,11]. Further analysis in a larger cohort of physicians is warranted to clarify the true relationship between ergonomics training and postural improvement.

The limitations of our study provide important context and highlight opportunities for future research. First, the intervention group began with higher baseline REBA scores, indicating worse posture and therefore a greater potential for improvement compared to controls. This imbalance was likely due to the small cohort size. Because the intervention group had higher baseline REBA scores, part of the observed improvement may reflect regression toward the mean. Although mixed-effects modeling adjusted for baseline differences, causal inference remains limited due to small sample size and baseline imbalance. Future studies with larger cohorts are needed to normalize baseline variation and increase statistical power. Second, resident posture varied widely due to differences in clinician characteristics (e.g., body size, age, sex, and experience) and patient anthropometrics (height and body habitus) [8-10]. Although gender distribution was reported, the study was not powered to evaluate sex-based ergonomic differences. Other factors, including patient volume, time of day, and MSK strain from surgical cases, may have contributed to additional variability. The potential Hawthorne effect, whereby residents may have altered posture due to awareness of being observed, could not be fully eliminated. Observing residents across an entire clinic session and blinding them to which encounters were analyzed likely minimized this effect. To account for these sources of variability and bias, we used a linear mixed-effects regression model that adjusted for baseline differences and accounted for clustering by resident. Future studies with larger, controlled, and stratified cohorts are needed to confirm these findings and to assess sex-based and patient-related ergonomic influences.

A further limitation relates to the study timeline. Post-intervention REBA scores were collected four weeks after completion of the ergonomics training. As a result, these findings reflect short-term postural improvements rather than sustained behavioral modification. As a pilot study, longer-term follow-up was beyond the scope of the current investigation. Future longitudinal studies with follow-up at six months or longer would be necessary to assess the durability of postural improvement and determine whether periodic reinforcement is required. The study lacked longer-term follow-up due to rotation logistics and the need to maintain a consistent clinical environment with standardized equipment across participants. Despite this limitation, the observed improvement suggests that targeted ergonomics education can have an immediate and measurable impact on resident posture [4,11].

The results support the feasibility and potential value of incorporating ergonomics education early in ophthalmology residency training, with immediate improvements in posture and potential occupational safety benefits that should be validated in longer-term studies. The intervention used in this study consisted of a brief, structured ergonomics training session focused on slit lamp positioning, operator posture, chair and equipment adjustment, and real-time corrective feedback. Beyond formal teaching sessions, future programs may benefit from practical reinforcement tools, such as point-of-care reminder cards, workstation checklists, or brief educational pamphlets placed in examination rooms to encourage consistent posture adjustment during daily clinical use. Future studies investigating ergonomics training curriculum in residency programs can build on our findings to evaluate retention of training, optimal timing for refresher sessions, and whether continuing medical education should include ergonomics as a formal requirement. Establishing an evidence-based, scalable ergonomics curriculum may help reduce short-term ergonomic risk for residents and warrants further investigation to determine long-term durability and impact on MSK outcomes [5,7].

## Conclusions

In this single-center quality improvement study, a brief, structured ergonomics education intervention was associated with significant improvements in posture among ophthalmology residents during SLEs, as reflected by reduced REBA scores. These findings suggest that targeted ergonomics training may meaningfully reduce short-term ergonomic risk during routine clinical activities in residency training. Importantly, the intervention was low-cost, feasible to implement within an existing residency program, and required minimal additional resources, supporting its practicality for broader adoption. Although this study was limited by a small sample size, short follow-up period, and baseline differences between groups, it provides objective evidence that early ergonomics education can positively influence resident posture. Given the high prevalence of work-related MSDs and their association with reduced productivity, disability, and early career attrition in ophthalmology, incorporating ergonomics training into residency curricula may represent a promising preventive strategy. Future studies should evaluate the efficacy of posture improvements over longer follow-up periods and assess the impact of repeated or longitudinal training. Additionally, research should determine the generalizability of this intervention across institutions and procedural specialties. Establishing standardized, evidence-based ergonomics education during residency may help promote occupational health, reduce MSK injury risk, and support longer, more sustainable careers for ophthalmologists.
